# Relationships between satisfaction with life, posttraumatic growth, coping strategies, and resilience in cancer survivors: A network analysis approach

**DOI:** 10.1002/pon.5948

**Published:** 2022-05-13

**Authors:** Matúš Adamkovič, Denisa Fedáková, Michal Kentoš, Miroslava Bozogáňová, Dominika Havrillová, Gabriel Baník, Mária Dědová, Ivana Piterová

**Affiliations:** ^1^ Institute of Social Sciences Centre of Social and Psychological Sciences SAS Košice Slovakia; ^2^ Institute of Psychology Faculty of Arts University of Prešov Prešov Slovakia; ^3^ Faculty of Humanities and Social Sciences University of Jyväskylä Jyväskylä Finland; ^4^ Department of Psychology Faculty of Arts Pavol Jozef Šafárik University Košice Slovakia; ^5^ Department of Psychology Faculty of Arts University of Trnava Trnava Slovakia

**Keywords:** bridge indicators, cancer, cancer survivors, coping strategies, network analysis, oncology survivors, posttraumatic growth, psycho‐oncology, resilience, satisfaction with life

## Abstract

**Objective:**

Cancer survivors' satisfaction with life should be seen through the psychological factors related to a person's capabilities to face and handle the situation. This study aimed to (1) examine the relationships of satisfaction with life, posttraumatic growth, resilience and coping strategies in a global network model, (2) find the bridge indicators between satisfaction with life and the other constructs, and (3) test for the invariance of the network structures across several moderating variables.

**Methods:**

In a heterogeneous sample of 696 cancer survivors (69% female; mean age = 53.1 ± 15.44 years; median time from being diagnosed = 4 years; breast cancer was the most frequent type of cancer) their satisfaction with life, resilience, coping strategies and posttraumatic growth was measured. In order to account for their complexity, the relationships between the constructs were explored using a network analysis approach.

**Results:**

The network analysis shows that satisfaction with life is strongly connected to resilience, moderately connected to coping strategies, and has a weak connection with posttraumatic growth. In the separate networks, the relationships between the psychological constructs were examined in greater detail. Besides some exceptions observed in the degree of disability, the networks were invariant across gender, age, years since being diagnosed, cancer type and treatment type.

**Conclusion:**

The findings suggest that interventions focused on cancer survivors' coping strategies and resilience could help increase their satisfaction with life. However, further replication of the proposed and/or modified model is needed.

## 1 BACKGROUND

Cancer causes significant changes to a patient's quality of life and subjectively‐experienced satisfaction with life (SWL). Moreover, it affects other psychological constructs which play an important role during and after treatment and should be considered in the process of health care provision.^1^

A cancer diagnosis is likely to increase psychological distress^2^ and could consequently trigger post‐traumatic reactions. However, these are not necessarily negative reactions and can result in an improvement in some areas of a survivor's life.^3^ SWL, post‐traumatic growth, resilience and coping strategies are defined as psychological constructs which have the potential for better life adjustment and even beneficial outcomes.^4^, ^5^, ^6^, ^7^, ^8^ A positive association between positive changes and life satisfaction among cancer survivors has been empirically supported.^4^, ^9^ *Satisfaction with life* represents the cognitive‐judgemental components of subjective well‐being. It is often considered synonymous with quality of life or as interconnected constructs.^10^, ^11^ Perceived satisfaction depends on the comparison of one's life circumstances with what is expected to be appropriate.^12^ In other words, the more positive changes persons who face a cancer experience, the more satisfied they are with their life.^11^ Reference to positive changes in a cancer survivor's life is often represented by *post‐traumatic growth* (PTG).^3^, ^4^, ^13^ PTG means development in various areas of life such as relating to others, new possibilities, personal strength, spirituality and appreciation of life and has been widely studied among cancer patients and cancer survivors.^4^, ^5^ Moreover, those with high PTG generally display higher psychological well‐being.^14^

While PTG leads to positive psychological changes following the struggle with a traumatic event and involves moving beyond pre‐trauma levels of adaptation, *resilience* of an individual has a protective potential^15^ and assumes the ability to bounce back and move forward with life after adversity. The level of resilience also plays an important role in the process of managing a cancer situation. Resilience is defined as the ability to withstand difficult circumstances^6^ and a positive relationship has been documented between resilience and quality of life.^7^ The level of resilience is also related to the coping strategies that a person uses to deal with a difficult situation. The use of active *coping strategies* is important in the process of resilience development. These are also known as solution‐oriented strategies or cognitive coping strategies (e.g., acceptance, attempt to reformulate the situation or a humorous approach) and can also result in increased SWL.^8^ Conversely, the use of avoidant coping strategies is not only associated with lower PTG, but also with a lower quality of life.^16^ It can be assumed that these constructs form an interconnected network of relationships. However, the constructs as well as the relationships between them could vary in cancer survivors due to several *moderating factors*. For example, a significant predictor of PTG is the time since the diagnosis.^3^ This might be because a certain distress period is needed for PTG to develop, if it ever does.^4^ A longer time since diagnosis is associated with the well‐being of survivors, with higher PTG levels^17^ and more effective coping with stress.^7^ In addition to the level of distress, age and gender also correlate with PTG in cancer patients.^18^ Regarding the demographic factors, gender, age and education are related to the resilience of cancer survivors. Among the illness‐related factors, resilience has been associated with time since diagnosis and presence of physical symptoms.^7^ In addition, the coping strategies of cancer patients were associated with demographic and illness‐related factors such as age, gender and stage of cancer.^19^

Most studies in psycho‐oncology have either focused on the bivariate relationships between SWL, PTG, coping strategies and resilience or tested mediation models. Indeed, there has been little attention paid to the interconnection of these variables.^7^, ^20^ Despite all four constructs repeatedly occurring in cancer studies, the outcome of our systematic review (see osf link: osf.io/tm49j/) has revealed that research examining the mutual relationships among them is missing. This has led the authors of the current study to investigate the mutual relationships between SWL, PTG, coping strategies and resilience in detail as well as to explore their structure in a sample of cancer survivors.

The aim of this study is to (1) explore and describe in detail the interconnection between these constructs (at the level of items/factors of the individual constructs), (2) identify which variables (bridge indicators) play the most important role in interconnecting these constructs, and (3) examine how the relationships between the constructs are moderated by variables such as gender, age, length since diagnosis of disease, degree of disability, type of cancer and method of treatment.

### 1.1 Network analysis and bridge indicators

The network approach to psychological constructs has become widely popular in recent years, especially in the field of psychopathology.^21^ In this approach, a psychological construct is not conceptualised as a latent variable causing the observable behaviour. Rather, the indicators of the construct are viewed as independent but mutually interacting entities and the construct emerges because of these indicators. The network approach thus allows one to study a selected construct in its complexity and reveal its structure and dynamics.^22^ As such, it shows what the relationships between the indicators are (conditional on all other indicators in the network), which indicators of the constructs are central/peripheral, as well as how well an indicator connects the other indicators in the network. In the network analysis, several constructs can be modelled within one network (of course, as in all other statistical models, the inference is only valid if the model involves all causally relevant variables). If a network involves several constructs, it might be of interest to study how these constructs are connected and see which indicators of the constructs are either (1) overlapping or (2) non‐overlapping but still play an important part in connecting the constructs.^23^ In psychopathology, such indicators are called bridge symptoms.^24^ In this paper, the term “bridge indicators” will be used as the research focuses on constructs that do not represent psychopathology. In a similar way to the established centrality/connectivity measures,^25^ the methods have been developed to examine how well an indicator connects two constructs within a network.^26^ Given the aims of this study, (1) a global network comprising SWL, PTG, coping strategies, and resilience will be estimated, (2) the networks of SWL and one of the other constructs will be estimated and the bridge indicators between SWL and the other construct examined in detail, and (3) the invariance of the estimated networks will be tested across several moderators.

## 2 METHODS

### 2.1 Participants and data collection procedure

Data from 696 cancer survivors (67% women; mean age = 53.1 ± 15.4 years) was collected throughout 2019 and 2020. Based on a recent systematic review,^27^ cancer survivorship is defined in this paper as a process that starts with a cancer diagnosis and continues throughout one's life. As such, the following inclusion criteria were applied: being 18 or older, having been diagnosed with cancer, not having a severe mental health or physical condition and not being terminally ill. Detailed information about the participants is available in Table 1. The participants were recruited in cooperation with the Slovak National Oncological Centre (NOU), oncological clinics and from cancer support groups. The ethical permissions were granted by the Ethical Committee at Trnava University (resolution no. 1/2018) and the National Cancer Institute (no. 13012020) and were subsequently approved by the management of each hospital. All participants provided written informed consent prior to participation. The research was carried out according to the Declaration of Helsinki.

**TABLE 1:** Demographic characteristics of participants (*N* = 696)

Variable	Percentage or mean ± SD or median
Gender (female)	66.5%
Age	53.1 ± 15.44
Partner status (married or in a relationship)	69.5%
Education ‐ high school	51.8%
Education ‐ university degree	31.6%
Employed	29.9%
Disablement pension	26%
Retired	32.2%
Years since diagnosis	4
Most frequent diagnosis ‐ breast cancer	30.3%
Time since finishing treatment (years)	2.95 ± 4.98
Experienced a relapse	19.3%
Undergone a combination of 3 or more types of medical treatment	35.9%
Attended cancer support groups	39.3%

### 2.2 Measures

The participants were administered the following psychological measures: *Satisfaction with life (SWL)* was measured using the Satisfaction with Life Scale (SWLS);^12^ *Posttraumatic growth (PTG)* was measured by the Posttraumatic Growth Inventory (PTGI);^28^ *Coping strategies (COP)* were measured by the Mini‐Mental Adjustment to Cancer Scale (Mini‐MAC)^19^; and *Resilience (RES)* was measured using the Brief Resilience Scale (BRS).^29^ Each of the measures has been used in previous studies with cancer survivors.^10^, ^11^, ^30^, ^31^ The measures were adapted to and administered in the Slovak language. In order to make the study easier to read, abbreviations are used when referring to items (lower case) or factors (upper case) of the corresponding constructs. More information about the measures is available at OSF link (https://osf.io/m3hzy/).

### 2.3 Statistical analysis

The missing data related to the scales (about 1%) were imputed using a regression‐based imputation method and the reliabilities of the measured scales and subscales were then calculated. As all the reliabilities were sufficiently high, sum scores were computed for the factors of the PTGI and Mini‐MAC This reduced the number of nodes in the network substantially while capturing the relevant aspects of the constructs, making the networks easier to interpret. As part of the descriptive process, the observed scores were compared with the scores obtained from different studies in the general Slovak population that had used the same measures. In order to answer the research questions, the following network models were estimated: a global network combining the items of the SWLS (5 nodes), the factors of the PTGI (5 nodes), the items of the BRS (6 nodes) and the factors of the Mini‐MAC (5 nodes). For a deeper understanding of which indicators are important in bridging SWL with each of the other constructs, the following were subsequently estimated: a) a network including the items of the SWLS and the items of PTGI; b) a network including the items of the SWLS and the items of BRS; and c) a network including the items of SWLS and the items of Mini‐MAC. To examine exactly how the constructs are interconnected, the average weight of all the edges was calculated (in their absolute values) connecting the two constructs. Additionally, a latent network model and a structural model, in which the correlations between the latent factors were allowed, were computed. The networks were estimated using the EBICglasso estimator. In order to reduce the possibility of finding spurious correlations, the tuning parameters were set to 0.50 to produce sparse networks. Given the aims of the research, the focus was specifically on the bridge variables and their centrality, following the method of Jones et al.^26^ Networks estimation performance and stability of the parameters was assessed. To examine how the networks differ across the subgroups (based on gender, age, time since being diagnosed, type of cancer, type of treatment and degree of disability), the networks for the subgroups were compared (a median split was used for the continual variables) using a Network comparison test.^32^ The analyses were performed in R, using *bootnet*,^25^ *NetworkComparisonTest*,^32^ and *networktools* ^26^ packages.

## 3 RESULTS

The descriptive characteristics of the scales including their reliability and comparison of the scores with the general Slovak population (the data were obtained from^33^), is available in Table 2. A bivariate as well as a partial correlation matrix of the items/scales can be found in the supplementary materials at https://osf.io/hfmaz/.

**TABLE 2:** Descriptive statistics and reliabilities of the scales

	M	SD	Potential range	Skewness	Kurtosis	*ω*_total_	Comparison with general population (Hedges' g)
SWL	4.46	1.34	1–7	−0.44	−0.42	0.90	0.22
RES	3.12	0.77	1–5	−0.10	−0.27	0.85	0.01
COP1	3.01	0.82	1–4	−0.81	−0.18	0.95	‐
COP2	2.69	0.74	1–4	−0.18	−0.61	0.90	‐
COP3	2.95	0.72	1–4	−0.72	0.11	0.80	‐
COP4	2.91	0.72	1–4	−0.55	0.03	0.81	‐
COP5	2.93	0.66	1–4	−0.62	0.09	0.78	‐
PTG1	3.07	1.25	0–5	−0.61	−0.36	0.92	0.70
PTG2	2.56	1.29	0–5	−0.09	0.83	0.88	0.29
PTG3	2.97	1.29	0–5	−0.45	−0.52	0.87	0.51
PTG4	2.53	1.71	0–5	−0.14	−1.26	0.89	0.48
PTG5	3.56	1.28	0–5	−0.95	0.30	0.86	0.81

Abbreviations: COP1 – COP5, factors of coping; PTG1 – PTG5, factors of posttraumatic growth; RES, resilience; SWL, satisfaction with life.

Given the complexity of the network approach, it is only the main findings that will be highlighted. Please note that the networks have had very good stability and accuracy. All the data, code and additional outputs are available in the supplementary materials at https://osf.io/9dsve/.

The first aim of the study was to explore and describe in detail the relationship between four psychological constructs. The network analyses revealed the following patterns: a global network comprising SWL, PTG, RES and COP has shown that the indicators of satisfaction with life are most strongly connected to the indicators of resilience, whereas there is virtually no connection between the indicators of PTG and satisfaction with life (see Figure 1). These results have been conceptually confirmed by the latent network model, in which the correlations between SWL, RES, COP, and PTG were 0.42, 0.27, and 0.12, respectively. In this global network, satisfaction with life (swl3), quick recovery after a difficult situation (res1), difficulty to cope with stressful situations (res2) and anxious preoccupation (COP4) served as the bridge indicators. The second aim of the study was to identify which variables (bridge indicators) play the most important role in bridging the four psychological constructs. When a closer look was taken at how SWL was connected with each of the other three constructs (in separate networks; see Figure 1A,B,C), the following results were observed: SWL and PTG were linked through personal strength (PTG3) and life acceptance (swl5). SWL and COP were mostly connected by life acceptance (swl5) and anxious preoccupation (COP4). Satisfaction with life (swl3) and life acceptance (swl5) were found to be the strongest bridge indicators linking SWL and RES. The third aim of the study was to identify how the relationships between the constructs are moderated by selected variables (e.g., gender, age, invalidity percentage, etc.). When the network invariance was tested across the moderating variables, it was found (with some exceptions in degree of disability), that the networks were invariant across gender, age, years since being diagnosed with cancer, degree of disability, cancer type and treatment type number. The exact results can be seen in Table 3.

FIGURE 1Visualization of the networks. (A) A global network of satisfaction with life, posttraumatic growth, resilience, and coping strategies. (B) A separate network of satisfaction with life and posttraumatic growth. (C) A separate network of satisfaction with life and coping strategies. (D) A separate network of satisfaction with life and resilience. swl and res, the items of the SWLS and BRS scales. PTG1, Relating to others; PTG2, New possibilities; PTG3, personal strength; PTG4, Spirituality; PTG5, Appreciation of life; COP1, Helplessness; COP2, Anxious preoccupation; COP3, Fighting spirit; COP4, Cognitive avoidance; COP5, Fatalism; for more information see the measures section. Solid lines indicate positive relationships, dashed lines indicate negative relationships. The thicker the line, the stronger the relationship. Bridge symptoms are in white ink. Names of nodes representing items are written in lowercase, names of nodes representing factors are written in uppercase

**Figure:**
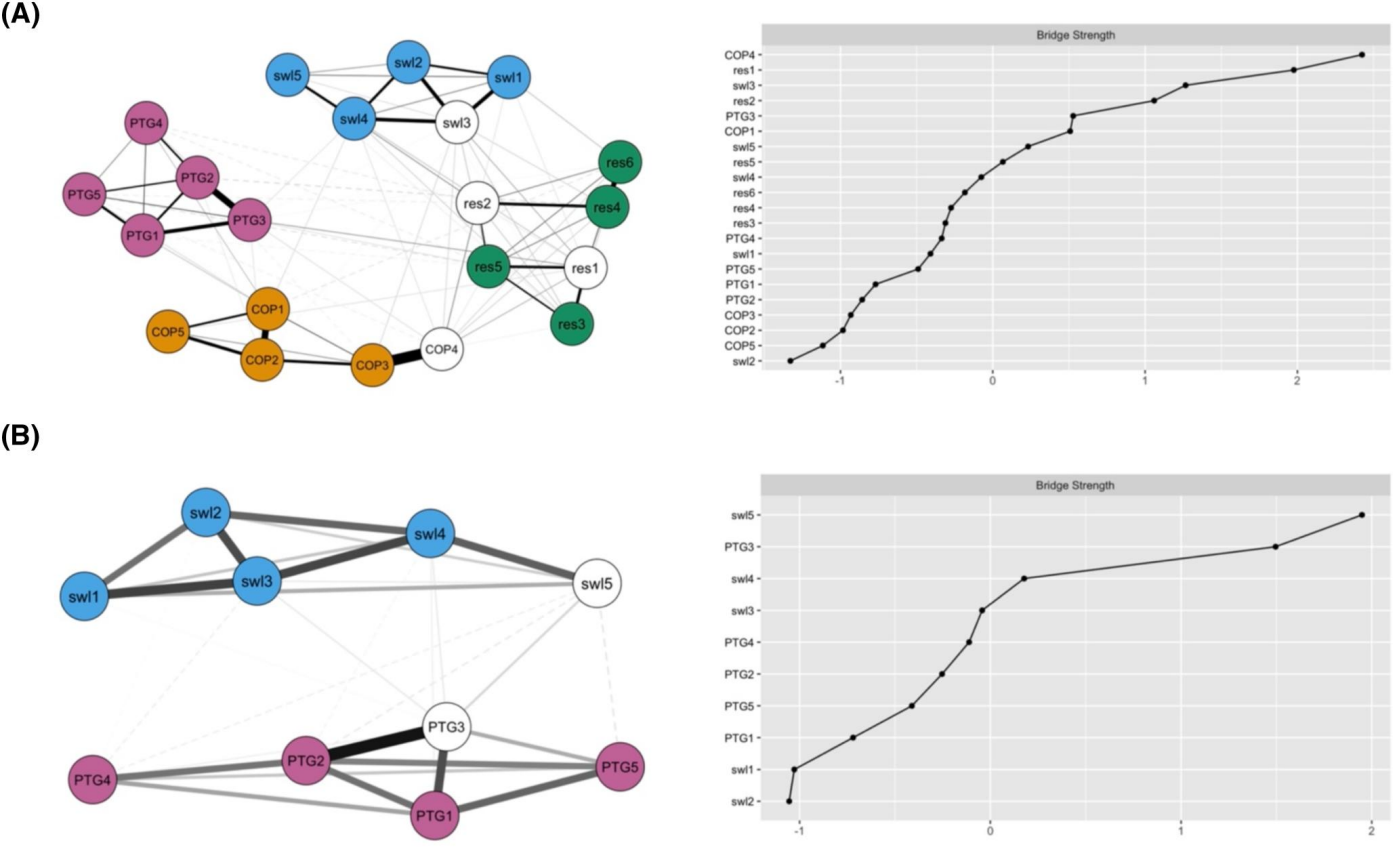

**Figure:**
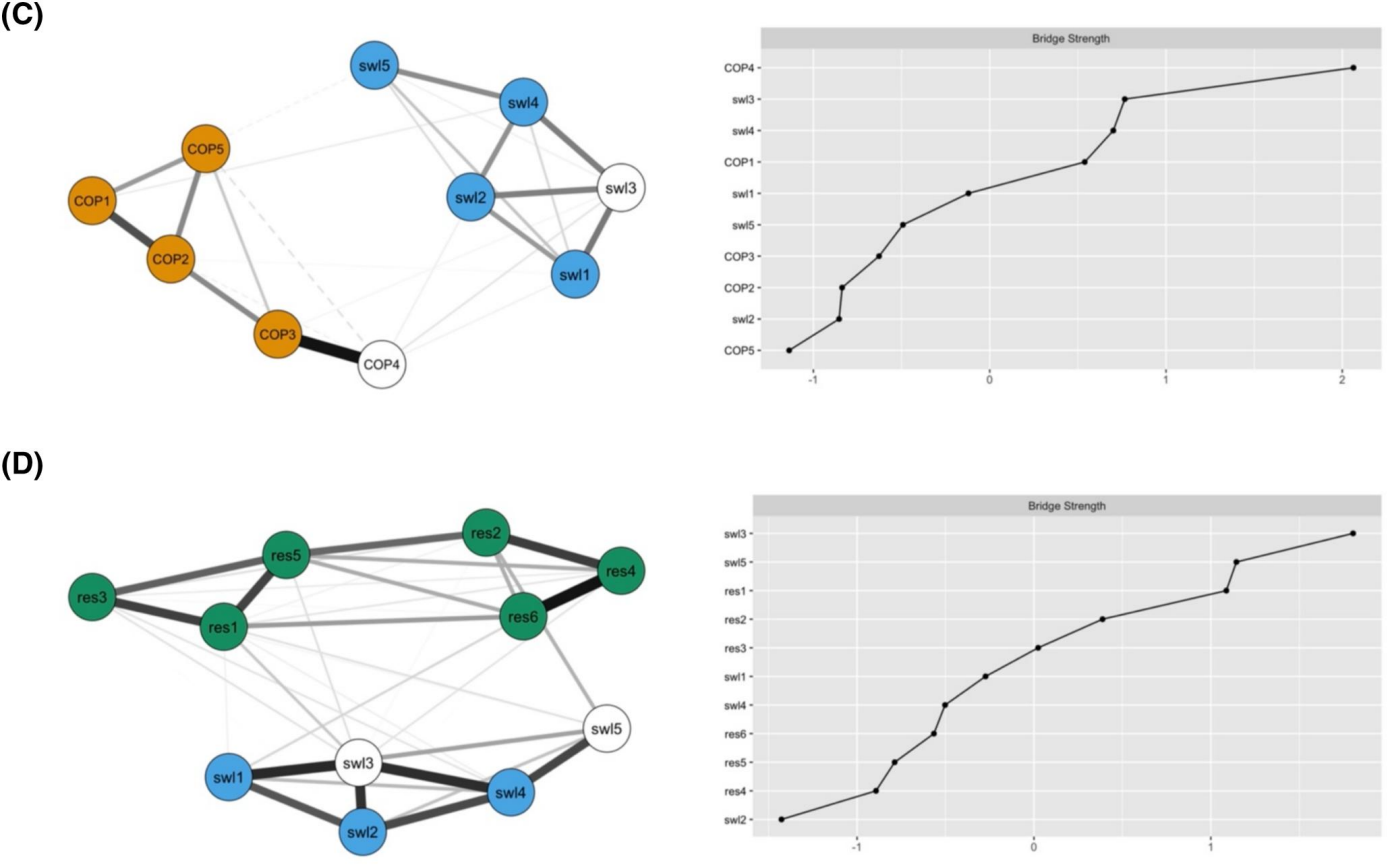

**TABLE 3:** Network invariance across the moderating variables

Moderating variable	Network	Network invariance test (*p*‐value)	Global strength invariance test (*p*‐value)
Gender (women; men)	SWL x RES	0.623	0.724
	SWL x COP	0.168	0.904
	SWL x PTG	0.510	0.600
Age (≤54; >54)	SWL x RES	0.553	0.346
	SWL x COP	0.294	0.147
	SWL x PTG	0.230	0.700
Years from diagnosis (<4; ≥4)	SWL x RES	0.643	0.438
	SWL x COP	0.359	0.135
	SWL x PTG	0.570	0.150
Degree of disability^a^ (<40%; ≥40%)	SWL x RES	0.233	0.016
	SWL x COP	0.023	0.923
	SWL x PTG	0.220	0.210
Cancer type (breast and ovarian; other)	SWL x RES	0.998	0.169
	SWL x COP	0.958	0.824
	SWL x PTG	0.980	0.180
Treatment (<3 types; ≥3 types)	SWL x RES	0.999	0.176
	SWL x COP	0.949	0.840
	SWL x PTG	0.970	0.230

Abbreviations: COP, coping; PTG, posttraumatic growth; RES, resilience; SWL, satisfaction with life.

^a^ as recognised by the evaluating committee of physicians.

## 4 DISCUSSION

This study has examined the mutual interactions and structures of four supportive/protective psychological constructs (SWL, PTG, RES and COP) among cancer survivors. The results of the network analyses have confirmed the expected relationships between the variables with the exception of the PTG and SWL connections. It was found that there were three items and one factor, satisfaction with life (swl3), quick recovery after a difficult situation (res1), difficulty to cope with stressful situations (res2) and anxious preoccupation (COP4), which were important in connecting the global network.

A significant relationship between SWL and RES has also been confirmed in other studies although this has been in different populations^6^, ^34^ or using a different measure.^35^ In this context, RES has been found to promote a long‐term positive attitude towards one's life. This is especially important because life satisfaction is affected by negative events and traumas throughout life which can be jeopardised if individuals have low resilience. SWL can also be used to assess whether individuals have bounced back from or shown permanent declines in their well‐being after experiencing adversity. The global network of the constructs implies the significant relationships of SWL and RES through items res1 and res2 which represent positively and negatively worded items. There is still a debate about the unitary factor structure of the BRS.^34^

Based on the presented results, destructive coping strategies appear to have a negative relationship with SWL. More specifically, SWL and COP strategies were linked by life acceptance (swl5) and anxious preoccupation (COP4). This finding is in line with the previous study in which anxious preoccupation was significantly negatively correlated with quality of life.^36^ Constructive coping strategies increased the quality of life (QoL), which is related to SWL.^37^, ^38^ However, they were not associated with SWL in the present study. Kershaw et al.^39^ have stated that avoidant strategies, such as behavioural disengagement and denial, may interfere with patients' and family caregivers' ability to problem‐solve in the face of advanced cancer. Bussel and Naus^37^ have found that a positive cognitive‐type of coping may be adaptive for cancer survivors. This result may hold implications for clinical practice as either decreasing destructive or increasing constructive coping strategies could be expected to have a positive effect on one's life satisfaction.

Although this study has not confirmed a direct relationship between PTG and SWL, other studies have shown a positive relationship between these constructs in cancer patients^11^ as well as in people living with HIV moderated by a period of treatment.^40^ Other studies have shown a relationship between a similar construct (QoL) and PTG in patients with cancer^8^, ^16^ or pointed to the ambiguity of results^41^ or/and dependence on the stage of the disease^13^, ^18^ or time since being diagnosed with cancer.^42^ The variation within the research sample (type and stage of cancer, time from being diagnosed, perception of a traumatic event, being part of a cancer support group etc.) seems to play a role and could affect the results. Based on the global network, PTG and SWL were indirectly linked through the level of resilience and coping strategies that the patient uses. Resilience may serve as a protective factor related to the use of active coping strategies and mediate its relationship with QoL.^15^ However, the role of resilience and coping strategies in the relationships between these variables requires further investigation. In terms of a separate network, SWL and PTG were bridged by personal strength (PTG3) and life acceptance (swl5) indicating that the presence of self‐reliance and personal strength is associated with the acceptance of life despite difficult life situations.

The observed relationships between the constructs were invariant across the entire spectrum of potentially moderating variables except for the degree of disability. Joshy et al.^43^ have provided evidence that QoL decreases with an increase in limitations to physical functioning. Previously mentioned study showed that physical disability is a key determinant of psychological distress and deteriorating QoL. The current findings support this claim as the degree of disability was found to be a significant moderator in the relationship between life satisfaction and coping strategies among cancer survivors. The potential differences in the observed relationships may stem from the cancer stage. However, such data was not available at our discretion.

### 4.1 Study limitations

The study has several caveats. (1) The present network analysis is of an exploratory nature and thus the results are needed to be replicated on other samples of cancer survivors. (2) The study has a cross‐sectional design and, as such, does not capture the intrapersonal dynamic of the relationships over time. (3) Given the heterogeneity of the sample, it is likely that the findings could be further shaped by certain factors which were not able to be controlled or were not focused on in the survey (e.g., the stage of cancer). However, the invariance testing indicates that the observed relationships were invariant across different moderating variables. Nonetheless, there is a dearth of studies on moderators of these relationships, and further research is welcome before an in‐depth discussion or conclusions can be offered. (4) The SWLS assesses the most general level of life satisfaction. For more precise inferences, it would be helpful to focus on the specific domains of quality of life. (5) The retrospective evaluation of PTG could be biased by social desirability or participants' motivation to perceive growth. The participants could thus report growth although this might not necessarily reflect the truth (reality/objective circumstances).^44^ (6) Other relevant variables related to psychological adaptation to cancer such as post‐traumatic stress or post‐traumatic depreciation (e.g.^45^) could be a subject of further examination within the network.

### 4.2 Clinical implications

Based on the observed relationships, there are several clinical implications for psycho‐oncological practice:

1. In general, knowing which nodes are central to a proposed network enables clinicians to tailor more effective intervention strategies (e.g.^46^).
2. Both SWL and PTG could be fostered by interventions that focus on the coping strategies adopted by cancer survivors. In particular, the interventions should aim to develop more constructive coping strategies, support a positive adjustment to the situation and reinforce the internal sources of coping.
3. Since RES serves as a protective factor for mental health (especially in stressful circumstances and at times of personal crisis), systematic training of it could also help to increase SWL and PTG.^47^

The QoL of cancer survivors could greatly benefit from having sufficient psychological therapy from psycho‐oncologists as well as from supportive survivor groups. For higher efficiency, psycho‐oncological support should ideally include the survivor's closest family and friends as well.^48^, ^49^, ^50^ Furthermore, psycho‐social support should continue even after the medical treatment is finished, although this is often absent in practice.

## 5 CONCLUSION

The present study offers a unique view of the structure and relationships between four psychological constructs ‐ satisfaction with life, post‐traumatic growth, coping and resilience ‐ that are highly relevant for understanding how cancer survivors fare in their situation. The results of the network analyses (both the global network as well as the separate networks) suggest that psychological interventions aimed at increasing the satisfaction with life of cancer survivors could benefit from focusing on the development of constructive coping strategies and reinforcement of resilience. Given the exploratory nature of this study, both further replications and studies with repeated‐measures designs are needed. Moreover, it would be beneficial to add other psychological constructs into the network analysis to deepen the understanding of the relationship complexity between them.

## CONFLICT OF INTEREST

None.

## Data Availability

The data that support the findings of this study are openly available in Open Science Framework at https://osf.io/9dsve/.
